# Determinants of Insomnia among Mothers during Postpartum Period in Northwest Ethiopia

**DOI:** 10.1155/2019/3157637

**Published:** 2019-04-01

**Authors:** Habte Belete, Eyaya Misgan

**Affiliations:** ^1^Psychiatry Department, College of Medicine and Health Sciences, Bahir Dar University, Bahir Dar, Ethiopia; ^2^Gynecology Department, College of Medicine and Health Sciences, Bahir Dar University, Bahir Dar, Ethiopia

## Abstract

**Objective:**

Postpartum period is a state of instability that may be accompanied by mood liability, anxiety, insomnia, and neuropsychiatric disturbance in women. This neuropsychiatric disturbance has a negative influence on the child's psychological and physical development. Our aim was to see the level of sleep difficulties among postpartum mothers in three obstetric care settings in Ethiopia.

**Method:**

Institutional based cross-sectional study was conducted at one referral hospital and two health centers. A total of 988 postpartum mothers had been interviewed for sleep difficulties by using Athens Insomnia Scale (AIS). Adjusted Odd Ratio (AOR) and 95% Confidence Interval (CI) were used and P-value <0.05 was used for indicating significant variables.

**Result:**

The prevalence of sleep difficulty between four to six weeks of postpartum period was 21.8% (215/988). Marital status of divorced/widowed/separated [AOR= 2.29, 95% CI (1.40, 6.08)], no educational opportunity [AOR= 2.35, 95% CI (1.57, 3.51)], having poor social support [AOR=2.82, 95% CI (1.63, 4.88)], alcohol use [AOR=1.58, 95% CI (1.13, 2.22)], history of depression [AOR=1.93, 95% CI (1.13, 3.31)], and who has poor support from husband [AOR=1.94, 95% CI (1.18, 3.18)] had association with sleep difficulty.

**Conclusion:**

There is a high magnitude of sleep difficulties during four to six weeks of postpartum period in postpartum mothers and they are associated with many preventable risk factors.

## 1. Introduction

Maternal mental health and related infants' developmental problems become the major reproductive health problems in low income countries. Postpartum period is a state of instability and is accompanied by mood liability, anxiety, insomnia, poor appetite, and irritability. Postpartum period has a link with the neurobiological mechanisms and there is elevation of monoamine oxidase, decrement of estrogen levels during the first week of postpartum period, and alteration of sleep pattern with other behavioral disturbances [1]. Circadian rhythms which are regulated by the body's internal master clock in the brain are influenced by this hormonal disturbance. Hormonal changes in the immediate postpartum period like decline of progesterone due to its sedative properties [2–4] and changes in melatonin levels [5] can affect circadian rhythms within the first 3 months and have been implicated in women's postpartum sleep difficulties.

Sleep difficulties during or before postpartum period are a serious maternal health issue which occurs at a crucial time in mothers' life and ends with various adverse effects on partners and the emotional, behavioral, and sleep difficulties of infants [6]. After giving birth, mothers are forced to awake at night to take care of their babies. This behavior is new and causes disturbance of natural sleep-wake cycle. The child's attachment with the mother and mother's behavioral health are influential to the child's psychosocial functioning in relation to the development of behavioral disturbances. Maternal sleep deprivation in the early weeks of postpartum period has a significant impact on infants' behavior [7, 8]. Mothers with sleep difficulties suffer from different disabilities which are associated with their infant's behavioral health. Most women experience significant sleep disruption, inadequate sleep, and high rates of symptoms of sleep disorder throughout their pregnancy period and this disturbance may be continued during their postpartum period [9]. Even compared with their late pregnancy, total sleep time and sleep efficiency deteriorated significantly from the delivery week to the 12^th^ postpartum week of their postpartum periods [10]. Many maternal related psychosocial factors determine their sleep health, and maternal mental illnesses also have a remarkable contribution for the occurrence of sleep difficulties [11, 12]. Maternal sleep disturbances have a strong negative outcome in maternal perceptions of the mother-infant relationship [13].

The magnitude of sleep difficulties during pregnancy is common. Different types of sleep problems can be raised during prenatal period such as symptoms of insomnia 57%, sleep-breathing difficulties 19%, and restless legs syndrome 24% [9]. The magnitude of sleep difficulties has reached up to 20% in early postpartum period [14], and wakefulness after sleep onset during postpartum period, 1^st^ postpartum week (21.1%), increased compared with late pregnancy (5.4%) [15]. The prevalence of sleep problem has reached up to 57.7% in seven weeks of postpartum period [16] and 52% in both pre- and postnatal periods [17]. There is a paucity study about sleep difficulties in low income countries and there is no information regarding the prevalence and determinant factors of sleep difficulties in developing countries especially in Ethiopia.

Obstetricians should give advice for mothers to make important adjustments in their sleep patterns during the postpartum period to avoiding the negative consequences of sleep disturbances such as dysphoric mood and impaired cognitive function [3]. For preventing sleep related behavioral problems in our setup, we have to address the magnitude and contributing factors of sleep difficulties, which is untouched in previous studies. Therefore, our objective was to see the magnitude of insomnia among postpartum mothers.

## 2. Methods

### 2.1. Study Setting

Facility based survey study was conducted from April to May 2017, at Felege Hiwot Referral Hospital, Bahir Dar, Ethiopia, and two (Bahir Dar and Hann) health centers in Bahir Dar town. Felege Hiwot Referral Hospital and two health centers are located in Northwest Ethiopia around 565 kilometers from Addis Ababa, the capital city of Ethiopia, having an elevation of 1,800 meters above sea level. The three health facilities located in Bahir Dar town and the town has a total population of 180,174; of these 93,014 are females. Currently, there are four hospitals (two public and two private), ten health centers, and a number of other private health institutions (clinics, pharmacies, and drug shops). Felege Hiwot Referral Hospital has postnatal care units at specialty level and serves for average of more than 1100 postpartum mothers per month and the two health centers also serve for an average of 500 mothers each per month.

### 2.2. Participants

All postpartum mothers who were attended at Felege Hiwot Referral Hospital and (Bahir Dar and Hann) health centers, in Bahir Dar town, for receiving obstetrical services were the source population. Study populations were postpartum mothers who were attending those facilities during data collection period. Postpartum mothers whose age was 18 years and above were included in the study and those who were unable to communicate due were not included in the study.

The number of samples required for the study has been calculated within single population proportion by taking 57.7% of the prevalence of sleep difficulties [16] with 3% of marginal error and 95% Confidence Interval and then total samples were 1,042. From those potential participants, 988 had completed the interview; but 20 denied participating; 19 failed to complete the interview and 15 were excluded. Systematic random sampling technique was used to select a total of 988 participants proportionally (494 from Felege Hiwot Hospital; 247 from Bahir Dar, and 247 from Hann health centers). Samples were selected within every two mothers (from each setting) and data was collected by diploma nurses by interviewing.

### 2.3. Instruments

Substance use was considered when mothers have consumed any social or illicit drugs at least once in the past 30 days [18]. Sleep difficulties were measured with Athens Insomnia Scale (AIS) that scored ≥ 6 points and which is unimportant instrument designed for quantifying sleep difficulty based on the 10^th^ revision of the International Statistical Classification of Diseases and Related Health Problems (ICD-10) criteria. AIS consists of eight items: the first five pertain to sleep induction, awakenings during the night, final awakening, total sleep duration, and sleep quality, while the last three refer to well-being, functioning capacity, and sleepiness during the day. AIS has a Cronbach's alpha of 90% [19]. Social support was assessed by using oslo-3 social support scale that consisted of three items [20]. Income was assessed by using relative income by leveling their own income as less than others, similar to others, and better than others. Education was categorized into uneducated (who were unable to read and write) and educated (who were able to read and write). Sociodemographic variables and clinical variables like comorbid medical illnesses were assessed by asking the participants if they were diagnosed for those illnesses before the survey. Depression among mothers was asked, if the mothers were clinically diagnosed with depression or not, and their chart also has been reviewed.

### 2.4. Analysis

After data was checked for completeness and consistency, it was entered by using Epi data version 3.1 and was analyzed by Statistical Package for Social Science version 20. Logistic regression analysis was implemented to identify factors associated with sleep difficulty. The strength of the association was presented by odds ratio with 95% Confidence Interval (CI). P value less than 0.05 was considered as statistically significant.

### 2.5. Ethical Guideline

Ethical clearance was obtained from Ethical Review Committee of College of Medicine and Health Sciences, Bahir Dar University. Formal permission letter was taken from administrative of the hospital and health centers. Written consent was taken from the participants. All participants who had insomnia were referred to clinic for better screening and management.

## 3. Results

### 3.1. Sociodemographic Characteristics of Mothers

A total of 988 postpartum mothers were participants in the survey and almost all 912 (92.3%) of them were from urban parts. The mean age of participants was 28.30 years (with standard deviation of 5.016 years). Among total mothers, 16.0% (159/988) has no educational opportunity (Table 1).

### 3.2. Clinical and Psychosocial Factors

Alcohol was a commonly used substance among mothers, 323/988 (32.7%). From the total mothers, 8.0% (79/988) had history of depression and 42/988 (4.3%) were using other substances during the survey (10 smoke cigarette, 16 chew khat, and 16 used cannabis).

### 3.3. Prevalence of Insomnia

The prevalence of insomnia was 215/988 (21.8%). From the total participants, 231/988 (23.4%) of them had reported that their child has got sickness frequently and of them 64/231 (27.7%) mothers had insomnia (Table 2).

### 3.4. Associated Factors with Insomnia

After multivariable regression, ineducation, marital status of divorced/separate/widowed, poor social support, history of depression, poor husband support, and alcohol drinking were significantly associated with insomnia (Table 3).

## 4. Discussion

Sleep difficulties during postpartum period have numerous serious behavioral disturbances for the mothers and their infants. Sleep difficulties have a negative impact on the attachment between the mothers and their infants. Maternal sleep difficulties have a strong negative effect on the affiliation of mothers and their infants [13].

In this study one in five mothers had insomnia. This proportion coincides with previously reported one at 1^st^ postpartum week wakefulness after sleep onset during postpartum period which was 21.1% [15]. However, this prevalence is lower than in Norway, 57.7% [16], which had been observed in the seven weeks of postpartum period and it is 52.0% in both pre- and postnatal periods in Michigan, USA [17]. Of course sample size difference, setting difference, sleep difficulties measurement difference, time frame for inclusion mothers after delivery, and other sociocultural variations may take the responsibility for this discrepancy.

Maternal sleep difficulty had a significant association with educational status and mothers who had no educational opportunity were found at more risk for sleep difficulties [AOR=2.35, 95% CI (1.57, 3.51)]. Maternal awareness about sleep hygiene and creating a good sleep habit may be mediated by the educational level of mothers and it is common that educated mothers have a better sleep hygiene habit.

Mothers who were currently unmarried had more than two times risk of insomnia than those married mothers [AOR= 2.29, 95% CI (1.40, 6.08)]. Being a mother is an important life event for most of mothers and means of happiness for the family. However, mothers may not be with their husband during these vital life events and may feel unlucky and develop sleep problems. Husband's support or care is important for all postpartum mothers and if they lost this support they might feel unfit to care for their child and get sleeplessness.

Insomnia had a significance association with mothers who had poor social support than good social support [AOR=2.82, 95% CI (1.63, 4.88)]. Mothers in Ethiopia are grown-up with intimate relatives and have good social support starting from their maternity leave to the first three to six postpartum months and even at least one family member must be always with them till ten days of their delivery period. However, some mothers may not get such advantageous and are at risk for sleep difficulties and related behavioral disturbances during their postpartum period [21].

Mothers who had drunk alcohol had a higher risk of insomnia than mothers who did not drink alcohol [AOR=1.58, 95% CI (1.13, 2.22)]. Alcohol use has a risk of sleep difficulties and other medical complications and also has impact on mothers' sleep during postpartum period. This finding was supported by earlier study where adolescents who drank alcohol were at more risk for sleep disturbances [22]. Mothers who had a history of depression were at more risk for insomnia than mothers who did not have depression [AOR=1.93, 95% CI (1.13, 3.31)]. This finding was supported by a follow-up study that states that mothers who had depression had more risk for sleep difficulties than mothers who did not have depression [11, 12]. Depression has a significant impact on their ability of care for their child and has a risk for sleep disturbances in their postpartum period. Mothers with history of depression may have mood instability, poor confidence, feeling of being unlucky, and low self-esteem which are risks for sleep difficulty.

Mothers who had poor support from their husband were at more risk for insomnia than mothers who had good support from their husband [AOR=1.94, 95% CI (1.18, 3.18)]. Husbands may encourage their wives by developing proactive strategies regarding how they help each other based on their own individual circumstances. Good husband support has remarkable prevention for sleep difficulties in postpartum periods [23]. Of course, poor support from husband has unpleasant thought among wives and this may lead mothers to think they are the only ones responsible for their child's care and they end up taking care of all activities which are risks for sleep disturbance.

## 5. Limitation

The study was in a single population and lacks comparative groups, which are limitations for generalization.

## 6. Conclusion

Proportion of maternal insomnia was found high in this study. Maternal insomnia has been associated with many preventable risk factors and we should pay attention to maternal sleep health during the postnatal maternal services. Future researchers should work on comparative studies with other groups of mothers in low-income countries.

## Figures and Tables

**Table 1:** Sociodemographic characteristics of the mothers in relation with sleep difficulties.

Characteristics	Insomnia		Overall	Chisq test (X^2^) (p-value)
	Yes	No		
*Age*				
18-24	50	152	202(20.4%)	2.6(.27)
25-34	124	492	616(62.3%)	
>34	41	129	170(17.2%)	
*Family size *				
1 to 2	16	30	46(4.7%)	11.6(.003)
3 to 5	175	695	870(88%)	
More than 5	24	48	72(7.3%)	
*Education*				
Uneducated	59	99	159(16.0%)	26.8(.000)
Educated	156	674	830(84%)	
*Religion*				
Orthodox	134	433	567(57.4%)	3.7(.29)
Protestant	25	122	147(14.9%)	
Muslim	52	207	259(26.2%)	
Catholic	4	11	15(1.5%)	
*Marital status*				
Married	160	646	806(81.6%)	26.4(.000)
Single	23	90	113(11.4%)	
Divorced/separate/widowed	32	37	69(7.0%)	
*Occupation*				
Has job	120	480	600(60.7)	2.8(.09)
Jobless	95	293	388(39.3%)	
*Relative wealth*				
Lower	70	232	302(30.6%)	3.9(.14)
Same	134	520	654(66.2%)	
Better	11	21	32(3.2%)	
*Living circumstance*				
With family	46	92	138(14.0%)	12.6 (.000)
Alone	169	861	850(86.0%)	

**Table 2:** Mothers' clinical and psychosocial factors in relation with sleep difficulties.

Characteristics	Insomnia		Overall	Chisq test (X^2^) (p-value)
	Yes	No		
*Mode of delivery*				
Cesarean section	94	345	439(44.4%)	.06(.81)
Vaginal delivery	121	429	549(55.6%)	
*Pregnancy*				
Planned	163	673	836(84.6%)	16.3(.000)
Unplanned	52	100	152(15.4%)	
*Comorbid medical illness*				
Yes	59	152	211(21.4%)	6.1(.014)
No	156	621	777(78.6%)	
*Alcohol use*				
Yes	94	229	323(32.7%)	15.2(.000)
No	121	544	665(67.3%)	
*Social support*				
Poor	89	118	207(21%)	70.0(.000)
Moderate	103	557	660(66.8%)	
Good	23	98	121(12.2%)	
*Frequently sick once child*				
Yes	64	167	231(23.4%)	6.3(.012)
No	151	606	757(76.6%)	
*Number of births*				
First	100	357	457(46.3%)	18.7(.000)
Second	66	149	315(31.9%)	
Third	24	133	157(15.9%)	
Fourth and above	25	34	59(6.0%)	
*History of depression*				
Yes	27	52	79(8%)	7.8(.005)
No	188	721	909(92%)	
*Good husband support*				
Yes	429	445	874(88.5%)	26.2(.000)
No	62	52	114(11.5%)	
*Feel confident to be good care-giver*				
Yes	143	617	760(76.9%)	16.8(.000)
No	72	156	228(23.1%)	

**Table 3:** Factors associated with insomnia in postpartum mothers.

Explanatory variables	Sleep difficulties		(Crude odd ratio)	(AOR) 95% CI
	Yes	No	(95% CI)	
*Family size*				
1 to 2	16	30	1:00	1:00
3 to 5	175	695	.47 (.25, .88)	1.64 (.67, 3.99)
>5	24	48	.94(.43, 2.04)	2.27(.71, 7.25)
*Living circumstance*				
Alone	46	92	2.01(1.36, 2.98)	.84(.50, 1.42)
With family	169	861	1:00	1.00
*Social support*				
Low	320	303	3.21(1.89, 5.46)	2.82 (1.63, 4.88) *∗∗*
Average	61	118	.78 (.48, .1.30)	.82(.48, 1.34)
High	110	76	1.00	1:00
*Alcohol use*				
Yes	219	104	1.84 (1.35, 2.59)	1.58(1.13, 2.22) *∗*
No	272	393	1:00	1.00
*Marital status*				
Married	160	646	1:00	1:00
Single	23	90	.97(.59, 1.58)	1.35(.79, 2.32)
Divorced/separate/widowed	32	37	3.38(1.75, 6.54)	2.29(1.40, 6.08) *∗*
*Education*				
Uneducated	59	99	2.57(1.78, 3.71)	2.35(1.57, 3.51) *∗∗*
Educated	156	647	1.00	1.00
*Number of births*				
First	100	357	.94(.67, 1.34)	.69(.36, 1.33)
Second	66	249	2.62(1.49, 4.60)	.62(.31, 1.21)
Third	24	133	.64(.39, 1.49)	.44(.21, .103)
Fourth and above	25	34	1.00	1.00
*Confident as good care-giver*				
Yes	143	617	.50(.36, .70)	.77(.52, 1.13)
No	72	156	1:00	1:00
*Frequently sick once child*				
Yes	64	167	1.54(1.09, 2.16)	1.06(.71, 1.59)
No	151	606	1:00	1:00
*Type of pregnancy*				
Planned	163	673	1:00	1:00
unplanned	52	100	2.14(1.47, 3.13)	.88(.54, 1.42)
*Comorbid medical illness*				
Yes	59	152	2.55(1.09, 2.19)	1.18(.79, 1.78)
No	156	621	1:00	1:00
*History of depression*				
Yes	27	52	1.99(1.22, 3.25)	1.93(1.13, 3.31) *∗*
No	188	721	1:00	1:00
*Good husband support*				
Yes	429	445	1:00	1:00
No	62	52	2.82(1.87, 4.25)	1.94(1.18, 3.18) *∗*

*∗*: significance at P value <.05; 1:00: reference; and *∗∗*: p value< .001.

## Data Availability

The datasets used and/or analyzed during the current study are available from the corresponding author on reasonable request.

## Abbreviations

AOR: Adjusted Odd Ratio
AIS: Athens Insomnia Scale
CI: Confidence Interval
USA: United States of America.
